# Epigenetic regulation and role of metastasis suppressor genes in pancreatic ductal adenocarcinoma

**DOI:** 10.1186/1471-2407-13-264

**Published:** 2013-05-29

**Authors:** Wolf Arif Mardin, Joerg Haier, Soeren Torge Mees

**Affiliations:** 1 Department of General and Visceral Surgery, University Hospital of Muenster, Albert-Schweitzer-Campus 1, 48149 Muenster, Germany; 2 Comprehensive Cancer Center Muenster, University Hospital of Muenster, Albert-Schweitzer-Campus 1, Muenster, 48149, Germany

**Keywords:** Pancreatic ductal adenocarcinoma, PDAC, Metastasis suppressor gene, Methylation, Epigenetics, Promoter

## Abstract

**Background:**

Pancreatic ductal adenocarcinoma (PDAC) is distinguished by rapid dissemination. Thus, genetic and/or epigenetic deregulation of metastasis suppressor genes (MSG) is a likely event during early pancreatic carcinogenesis and a potential diagnostic marker for the disease. We investigated 9 known MSGs for their role in the dissemination of PDAC and examined their promoters for methylation and its use in PDAC detection.

**Methods:**

MRNA expression of 9 MSGs was determined in 18 PDAC cell lines by quantitative RT-PCR and promoter methylation was analyzed by Methylation Specific PCR and validated by Bisulfite Sequencing PCR. These data were compared to the cell lines’ *in vivo* metastatic and invasive potential that had been previously established. Statistical analysis was performed with SPSS 20 using 2-tailed Spearman’s correlation with P < 0.05 being considered significant.

**Results:**

Complete downregulation of MSG-mRNA expression in PDAC cell lines vs. normal pancreatic RNA occurred in only 1 of 9 investigated genes. 3 MSGs (CDH1, TIMP3 and KiSS-1) were significantly methylated. Methylation only correlated to loss of mRNA expression in CDH1 (P < 0.05). Bisulfite Sequencing PCR showed distinct methylation patterns, termed constant and variable methylation, which could distinguish methylation-regulated from non methylation-regulated genes. Higher MSG mRNA-expression did not correlate to less aggressive PDAC-phenotypes (P > 0.14).

**Conclusions:**

Genes with metastasis suppressing functions in other tumor entities did not show evidence of assuming the same role in PDAC. Inactivation of MSGs by promoter methylation was an infrequent event and unsuitable as a diagnostic marker of PDAC. A distinct methylation pattern was identified, that resulted in reduced mRNA expression in all cases. Thus, constant methylation patterns could predict regulatory significance of a promoter’s methylation prior to expression analysis and hence present an additional tool during target gene selection.

## Background

Pancreatic ductal adenocarcinoma (PDAC) is a highly aggressive malignancy with a dismal 5-year survival rate of less than 5%, a median survival of 6 months and a mortality to incidence ratio of 0.98 [1,2]. While surgical intervention represents the only curative treatment option, more than 80% of patients with PDAC present with irresectable disease [3,4]. Yet even after surgery, 5-year survival in specialized centers reaches no more than 25% with (neo)adjuvant treatment and remains below 15% without it [5,6]. Existing strategies seem to be exhausted and novel markers as well as new therapeutic targets are needed to both move forward the time of diagnosis and increase treatment efficacy.

Genetic alterations to factors and pathways underlying pancreatic ductal carcinogenesis, such as KRAS, TP53, SMAD4 and Hedgehog are under close investigation [7]. However, beside genetic alterations, epigenetic mechanisms for gene inactivation, such as transcriptional silencing by promoter methylation, seem to be important in the pathogenesis of PDAC [8]. Recent studies have shown that aberrant methylation of CpG-islands is a common mechanism associated with the silencing of tumor-suppressor and cancer-related genes in pancreatic cancers [9], among them BRCA1, APC, and p16INK4a [10-12]. These aberrant methylation patterns and the resulting changes in gene expression may over the long term be drawn upon as therapeutic targets. A more tangible use however may be their exploit as biomarkers; these would ideally enable earlier diagnosis or even screening for pancreatic cancer. Presently, the disease is mostly diagnosed by the onset of clinical symptoms which occur at later stages, when a curative approach, i.e. resection, is mostly no longer possible. Moving forward the time of diagnosis would thus increase survival through identification at more treatable disease stages [13,14]. As a consequence, biological changes used for the detection of PDAC should ideally be present early on in pancreatic carcinogenesis and precancerous lesions. Prevalence of low-level aberrant methylation has indeed been detected in well-defined pancreatic intraductal neoplasia (PanIN) lesions and overexpression of DNA methyltransferase 1 has been found in precancerous conditions and it increased with disease progression [15]. Detection of aberrant methylation in body fluids such as serum, urine, pancreatic juice or sputum could also be a useful marker for PDAC [16]. For example, analysis of NPTX2 [17,18], RASSF1A [19], cyclin D2 [20], ppENK and pi6 [21] hypermethylation may enable the creation of a highly sensitive and less invasive panel of markers for pancreatic cancer. These findings raise hopes that early markers of PDAC may be developed based on aberrant methylation patterns.

Due to the disease’s rapid progression and early metastasis formation, metastasis suppressor genes (MSGs) may be deregulated early during pancreatic carcinogenesis. Clinically and experimentally, primary tumor development and metastasis formation are distinct processes. Metastasis suppressors are defined as inhibitors of metastasis at any step of the metastatic cascade without interfering with primary tumor growth [22]. The discovery of these endogenous molecules that exclusively inhibit metastasis and the understanding of their actions suggest that metastasis is an amenable therapeutic target. However, only few of the currently known MSGs have been investigated in pancreatic cancer so far. In the present study, we selected a comprehensive panel of MSGs based on review of metastasis suppressors by Shevde and Welch [23].

Taken together, our review of the current literature revealed 1) earlier diagnosis of PDAC would increase survival, 2) hypermethylation occurs early during pancreatic carcinogenesis and 3) metastasis suppressor genes have not been sufficiently researched in this context. Accordingly, we investigated a number of known MSGs for their influence on PDAC dissemination and the methylation status of their promoter region In order to identify possible PDAC biomarker candidates.

## Methods

### Ethics statement

All animal experiments were conducted in accordance with the national guidelines for the care and use of laboratory animals, and the experimental protocol was approved by the state agency for animal welfare of North Rhine-Westphalia (LANUV; NRW, Germany).

### Genes of interest

Analyses were carried out for 9 known MSGs, as reviewed by Shevde and Welch [23]: BRMS1, CD82, CDH1, KiSS-1, MAP2K4, MED23, NDRG1, TIMP3 and TXNIP.

### Cell lines

18 human PDAC cell lines were analyzed in vitro. All cell lines were obtained from American Type Culture Collection, except A818, PT45, HPAF2 and MiaPaCa2 which were originally obtained from American Type Culture Collection and provided by H. Kalthoff, Department of Surgery, University Hospital Kiel, Germany and H. Hotz, Department of Surgery, Charite CBF, University Hospital Berlin, Germany. Cells were maintained in recommended growth media, and all media were supplemented with 10% heat inactivated fetal bovine serum (Gibco / Invitrogen, Karlsruhe, Germany) and were mycoplasma negative. For culturing, they were incubated at 37°C in humidified air with 5% or 10% CO2. The medium was replaced twice a week, and cells were maintained by serial passaging after trypsinization with 0.1% trypsin.

### Animal model and biological classification of cell lines

An orthotopic implantation tumor model using four-week-old male nude mice (Crl:NU/NU-nuBR) had been carried out previously [24]. In brief, four-week-old male nude mice (Crl:NU/NU-nuBR) were injected subcutaneously with each human PDAC cell line. These mice were euthanized after 3 to 4 weeks and the donor tumors collected, fragments of which were inserted orthotopically into another mouse’s pancreatic parenchyma (n = 10 mice per cell line). HS766T and PL45 failed to form tumors in donor mice and thus were excluded from *in vivo*, but not from *in vitro* analysis. After a growth phase of 12 weeks, primary tumor volume, local infiltration and patterns of local and systemic metastases were assessed systematically as previously described [25]. The results were compiled into a score each for metastasis and invasion.

### Nucleic acid preparation

DNA and RNA extraction from cell lines was performed using the DNeasy Blood & Tissue and the RNeasy Mini Kit from Qiagen (Hildesheim, Germany) according to the manufacturer’s specifications. A total RNA preparation from human pancreas was acquired from Applied Biosystems (Darmstadt, Germany). RNA samples were stored at -80°C. CDNA was generated using the High Capacity cDNA Reverse Transcription Kit from Applied Biosystems according to the manufacturer’s specifications. CDNA was stored at -20°C. Purity and concentration of nucleic acids were measured in a biophotometer (Eppendorf, Hamburg, Germany).

### Bisulfite modification

Bisulfite modification of DNA was performed with EpiTect Bisulfite Kits from Qiagen according to the manufacturer’s specifications. Obtained products were stored at -20°C.

### Methylation assays

Methylation specific PCR (MSP) and Bisulfite sequencing PCR (BSP) were carried out as previously described for in vitro cell lines [26]. BSP results were analyzed with the Beckman Coulter CEQ 8800 Genetic Analysis System software v9.0 using C- to T-peak ratios to define a CpG-dinucleotide as methylated, unmethylated or heterogeneously methylated for each CpG-dinucleotide. Point values were assigned to each CpG-dinucleotide according to its methylation status as follows: unmethylated: 0; heterogeneously methylated: 1; methylated: 2. A methylation-score was calculated for each gene in each cell line by using the average point value of all investigated CpG-dinucleotides for that gene, resulting in values from 0.0 (completely unmethylated) to 2.0 (completely methylated).

### Quantitative reverse transcriptase-PCR

Quantitative reverse transcriptase-PCR (qRT-PCR) had been previously performed for in vitro cell lines [26]. Calculations were carried out using the qBase algorithm in Microsoft Excel using the 2^-ΔΔCT^ Method with PPIB and HPRT1 as reference genes [27].

### Statistical analysis

Statistical analysis was performed using 2-tailed Spearman’s correlation. 95% confidence intervals were calculated and P < 0.05 was considered significant. SPSS 20.0 (SPSS Inc., Chicago, IL, USA) was used for these calculations.

## Results

### Animal model: tumor biology of the PDAC cell lines

*In vivo* metastatic potential had been previously investigated for 16 PDAC cell lines and quantified by a metastasis- and invasion-score [24]. In brief, the scores were calculated by crediting one point for every local infiltration, every colonized organ, and for multiple metastatic lesions per organ. Score values represent mean sums of the obtained credit points for all mice in a group.

### Metastasis suppressor gene mRNA expression

We investigated the expression of 9 MSGs by qRT-PCR for 18 PDAC cell lines and normal human pancreatic RNA. Gene selection was performed at the beginning of the investigation and reflects the known MSGs at that time. In 6/9 genes (BRMS1, CD82, CDH1, MED23, NDRG1 and TXNIP) and both reference genes (HPRT1, PPIB) mRNA was detected in all samples. Of the remaining 3 genes, KiSS-1 was detected in all cell lines except MiaPaCa2; MAP2K4 was detected in all cell lines except A818, MPanc 96, PaTu 8902, PaTu 8988S and PaTu 8988 T; TIMP3 was detected in all cell lines except A818, MiaPaCa2, PaTu 8902 and PaTu 8988 T.

**Figure 1:**
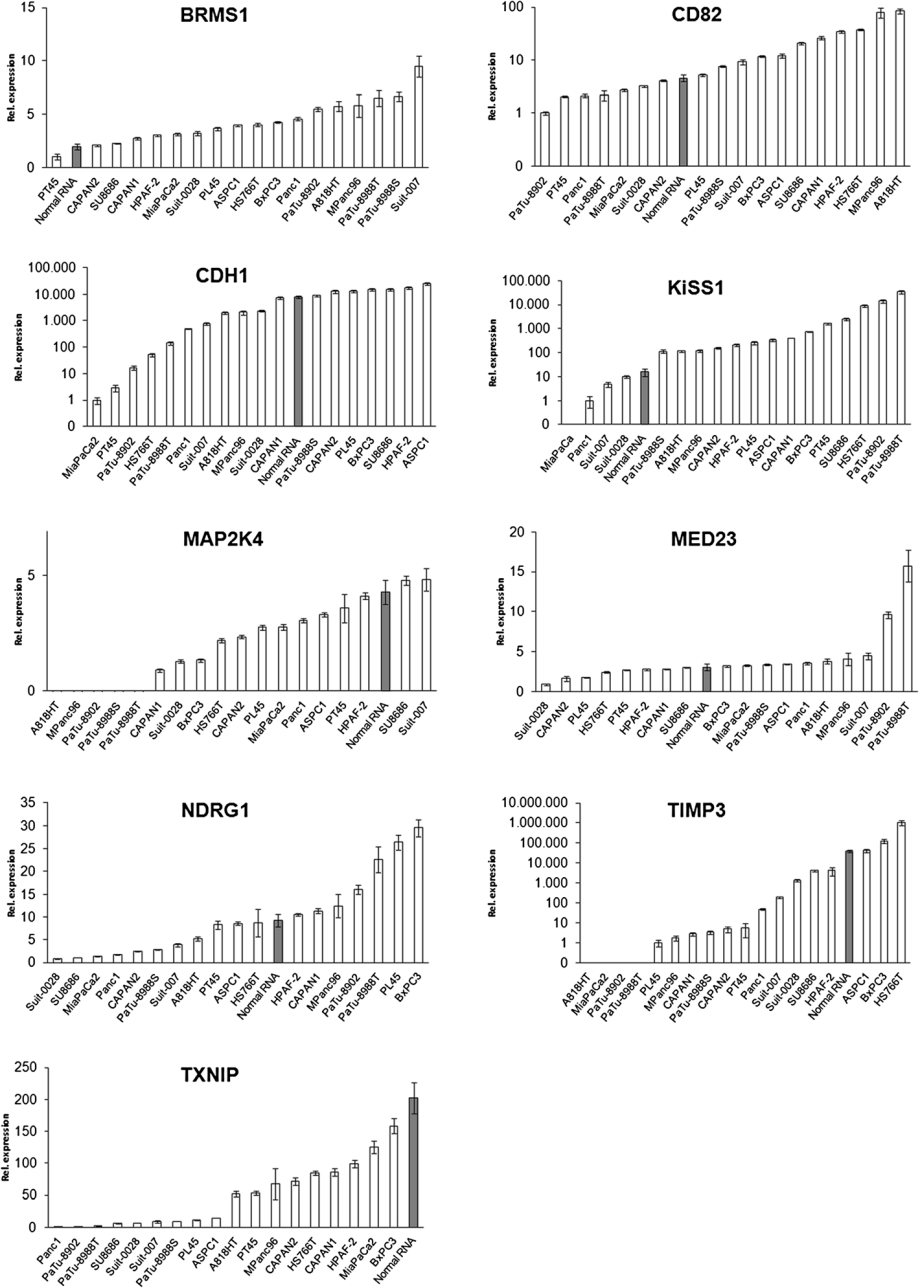
**Metastasis suppressor gene mRNA expression. **Legend: MRNA expression (qRT-PCR) of metastasis suppressor genes in PDAC cell lines and normal pancreatic RNA as previously determined by our group [26]. Normal pancreatic RNA is highlighted, demonstrating that downregulation of metastasis suppressor genes in PDAC cell lines vs. normal RNA was not a uniform occurrence.

Of note: Isolation of in vivo samples of sufficient quality (RIN > 7.0; tumor cells >80% in microscopic analysis) was not possible. The underlying reasons are presented in the discussion section.MAP2K4, TIMP3 and TXNIP were downregulated in most PDAC cell lines while BRMS1 and KiSS-1 showed predominant upregulation vs. normal pancreatic RNA (Figure 1 and [26]).

### Methylation specific PCR

**Figure 2:**
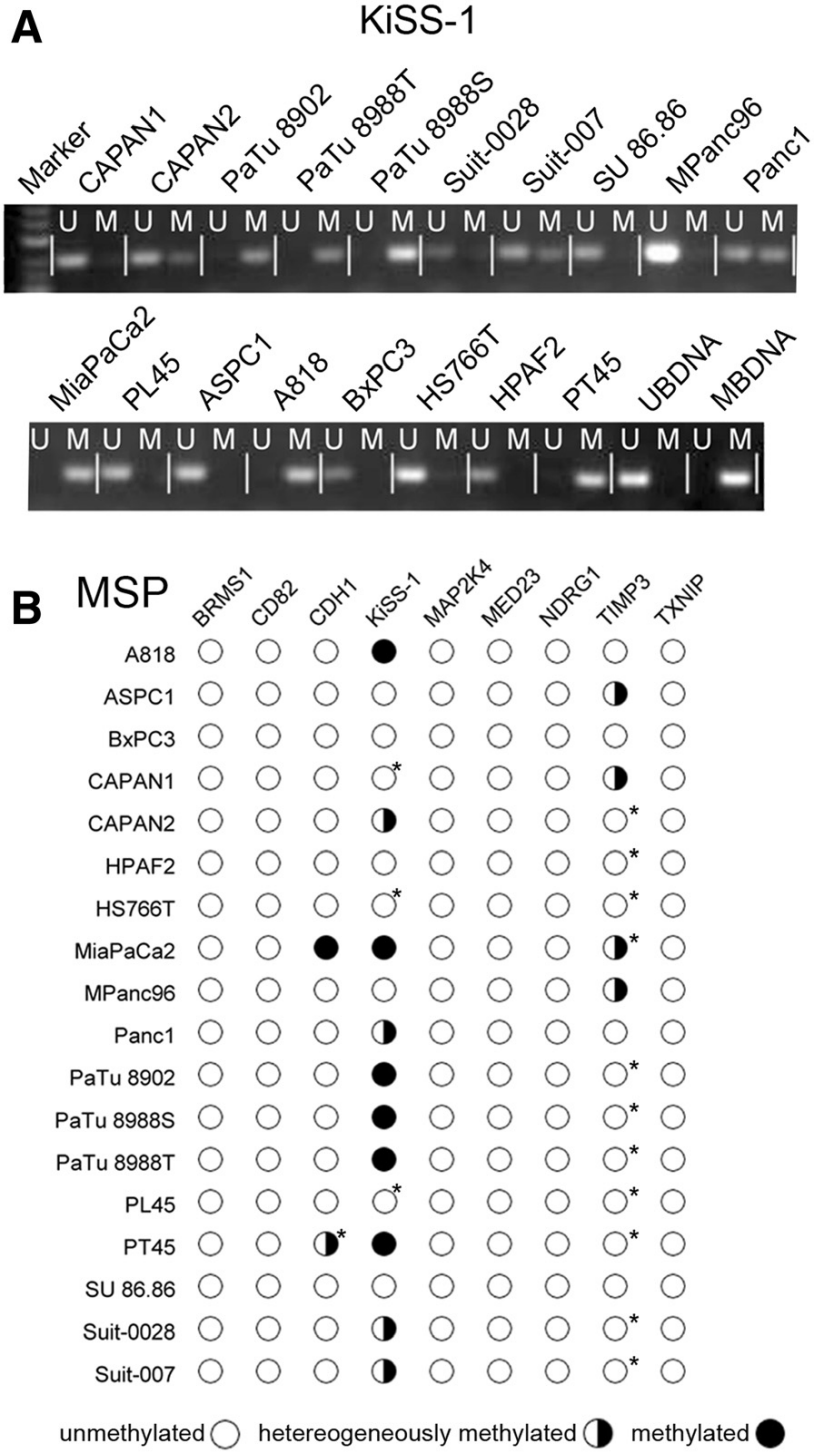
**Methylation specific PCR. **Legend: **A**) Example of a methylation specific PCR: *KiSS-1*. Samples are noted above the image and primer specificity is declared as U, specific for unmethylated template, or M, specific for methylated template. 3 distinct signal patterns were observed: either only the U- or the M-band was present, or both bands were present. Cell lines were thus identified as unmethylated (e.g. MPanc96), methylated (e.g. PaTu8988S) or heterogeneously methylated (e.g. CAPAN2). **B**) MSP-analysis for all cell lines and genes. Each column represents a gene and its methylation status in a cell line is indicated by partially filled circles. Methylation status for each cell line was determined as illustrated in Figure 3A for the example for KiSS-1. *different results for MSP- vs. BSP-analysis (compare to Figure 3).

MSP analysis of CpG-islands in the promoter region of the investigated genes showed 3 patterns. 1) and 2): Only one band was found per MSP: either the PCR with the methylation-specific primer showed a band while the PCR with the unmethylation-specific primer did not or vice versa. 3) Both PCRs from the same MSP showed bands simultaneously. The results were therefore classified as homogenously methylated, homogenously unmethylated or heterogeneously methylated (Figure 2A). No gene was methylated in all cell lines while several genes were unmethylated across all cell lines (Figure 2B). Gel pictures are provided in Additional file 1.

### Bisulfite sequencing PCR and direct sequencing

**Figure 3:**
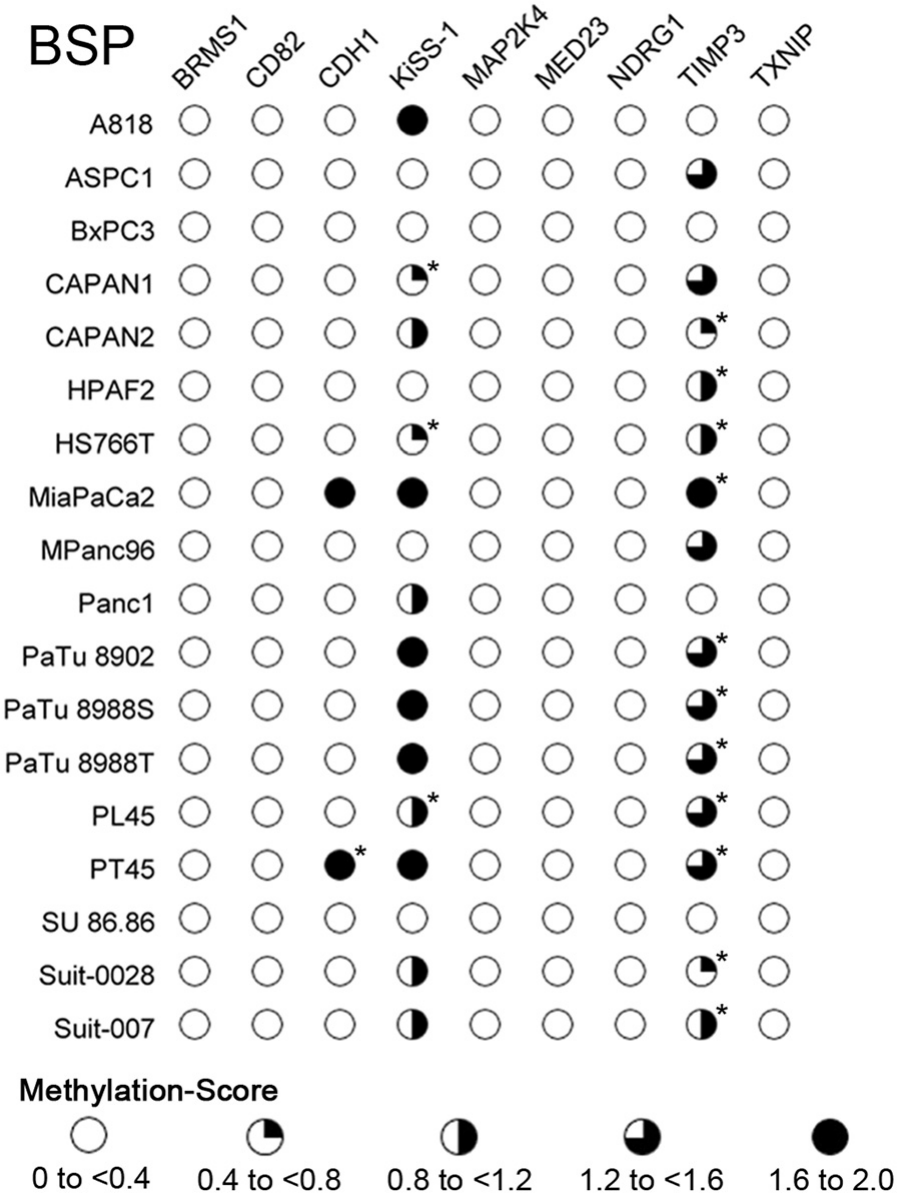
**Overview of bisulfite sequencing methylation scores. **Legend: Increasingly colored in circles represent methylation score ranges from bisulfite sequencing. Residual methylation was present in BRMS1, which however still translated into an overall methylation score of <0.4. Detailed methylation maps are provided in Figure 4.

**Figure 4:**
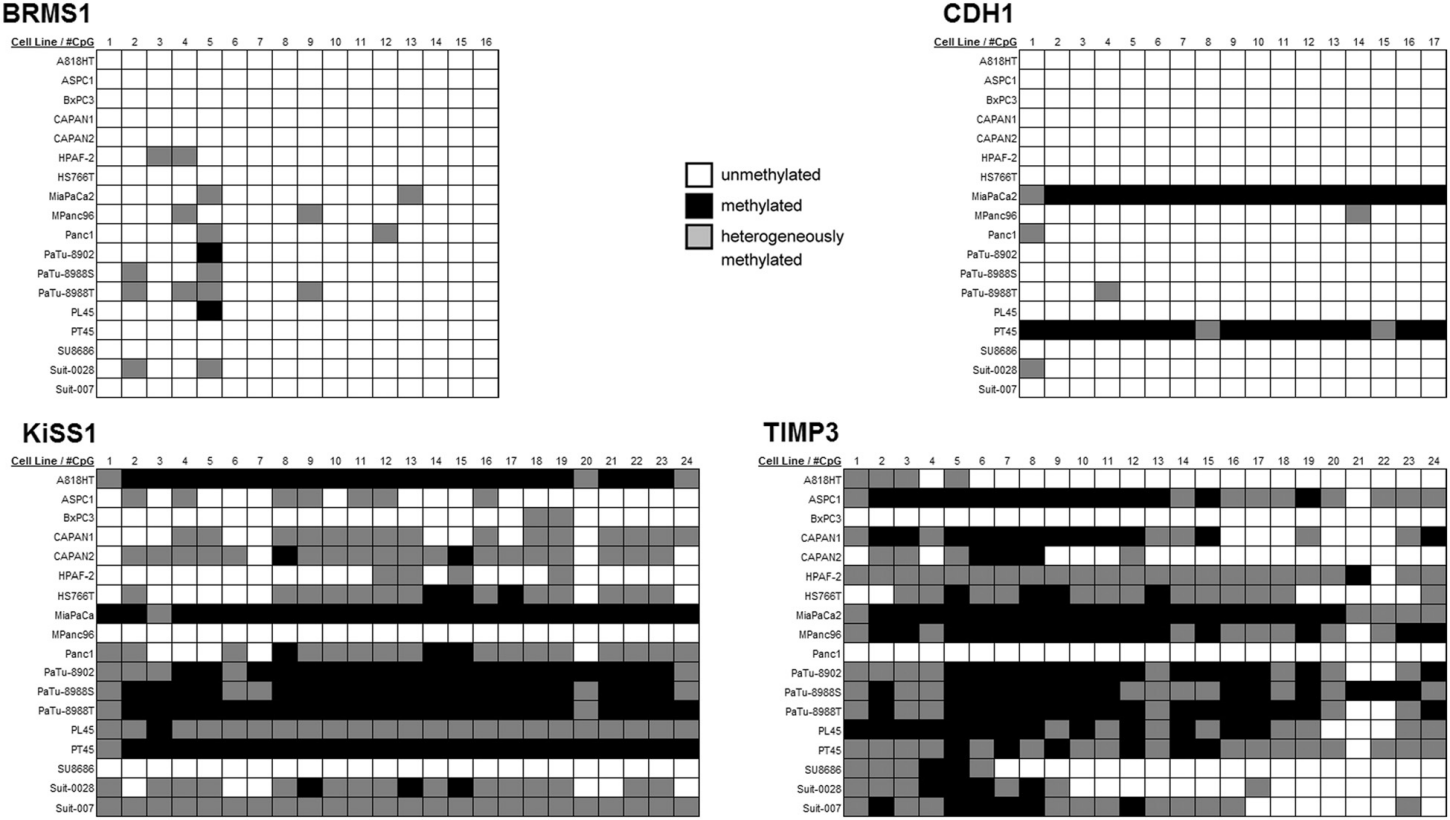
**Methylation volatility. **Legend: Each box represents one sequenced CpG in 5′ to 3′ order whose methylation status is indicated by coloration. Some cell lines have uniform methylation patterns throughout the entire investigated region of a gene (e.g. CDH1 in MiaPaCa2: constant methylation pattern), while a more inconsistent pattern is present in other cases (e.g. PL45 in TIMP3: variable methylation pattern).

Three types of signals were present at the CpG-sites: 1) A T-peak and no C-peak; classified as an unmethylated CpG-dinucleotide 2) A C-peak and no T-peak; classified as a methylated CpG-dinucleotide 3) Superimposing C- and T-peaks; classified as heterogeneously methylated CpG-dinucleotide. 5 of the 9 investigated genes (CD82, MAP2K4, MED23, NDRG1, TXNIP) showed no methylation throughout the entire sequenced region (Figure 3). The remaining 4 genes, BRMS1, CDH1, KiSS-1 and TIMP3 showed significant methylation, with differing methylation patterns and intensities (Figure 4).

### Correlation between MSP and BSP-direct sequencing

MSP-status and the BSP methylation-score were highly correlated in the methylated genes (P < 0.005, except TIMP3: P < 0.05). However, differing results between MSP and BSP-direct sequencing were discovered for 2 cell lines in TIMP3 and 11 cell lines in KiSS-1. In all of these instances, BSP-direct sequencing was more sensitive to methylation than MSP (Figure 2B and Figure 3). The unmethylated genes were identified as such by both methods with only BRMS1 showing minor methylation in BSP-direct sequencing.

Overall, BSP proved more sensitive to methylation than MSP, delivering an explanation for the only slightly differing results in KiSS-1. In TIMP3 however, greater discrepancies were encountered, suggesting the possibility of differential methylation patterns within the promoter island as the BSP-sequenced region did not include the region in which the MSP primers were situated.

### Methylation patterns

**Figure 5:**
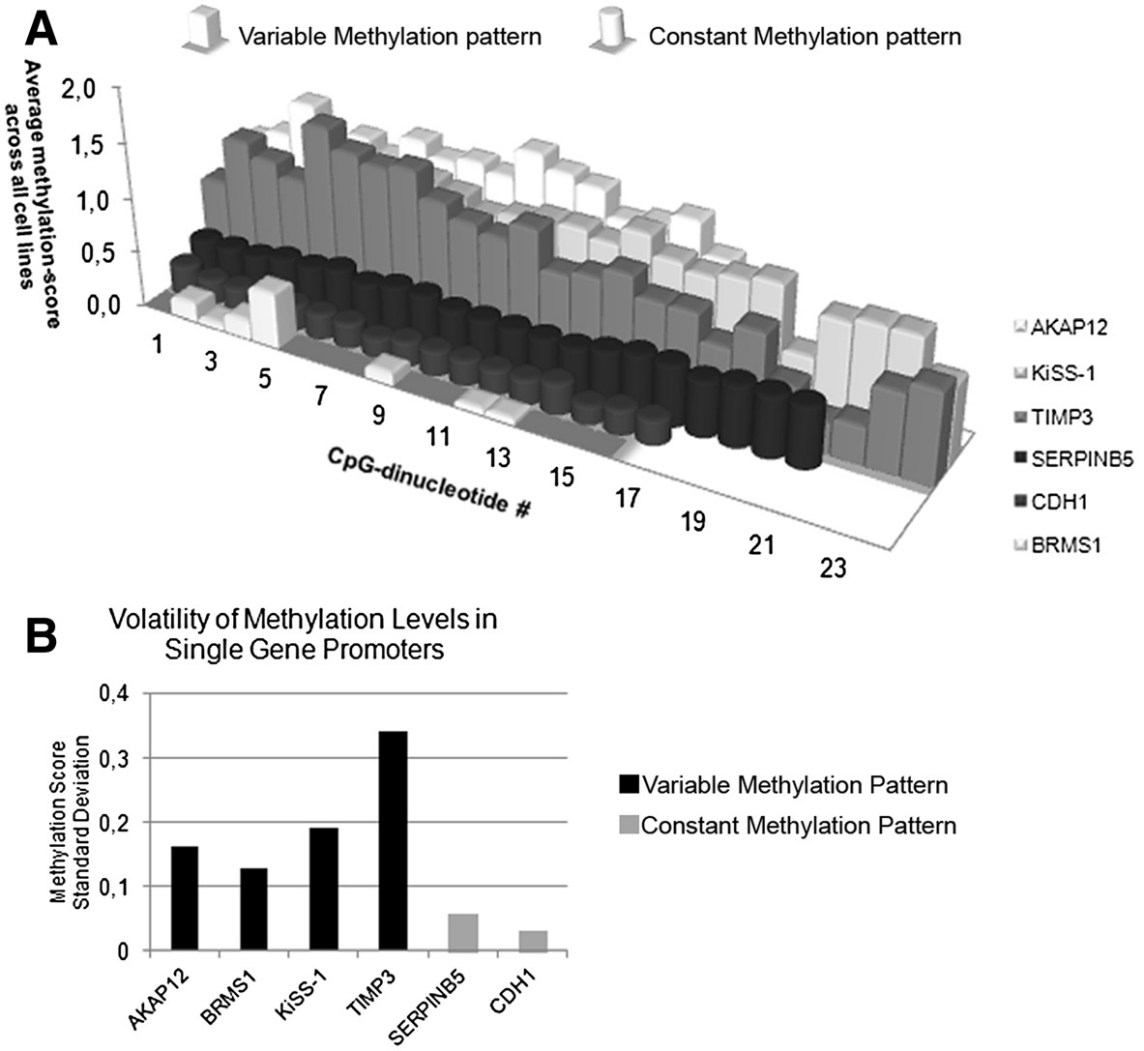
**Methylation volatility. **Legend: **A**) Each row represents the sequenced CpG sites of one gene with each bar standing for one CpG-site in 5′ to 3′ direction. Bar height denotes the average methylation-score of that CpG-site across all cell lines. Variably methylated genes (*AKAP12, BRMS1, TIMP3*, *KiSS-1*) showed generally higher methylation with greater volatility than genes with constant methylation (*CDH1*, *SERPINB5*). **B**) The observation in A is quantified by differing levels of methylation volatility: genes with constant methylation had lower standard deviation of methylation levels than those with variable methylation.

We identified two distinct methylation patterns in the four genes with significant promoter methylation. The first pattern was dubbed variable methylation: In BRMS-1, KiSS-1 and TIMP3, volatility of methylation was high with parallel occurrence of methylated, heterogeneously methylated and unmethylated CpGs at a single methylation site (Figure 4). The second pattern, present in CDH1, was termed constant methylation: CDH1 was only methylated in 2/18 (11%) cell lines. In contrast to the methylation-variability observed in the first group, the CDH1 promoter had a constant methylation pattern for single cell lines: the cell lines displayed either uniformly high or uniformly low methylation across the entire sequenced promoter region with low methylation volatility. Promoter methylation and mRNA expression data from 2 additional metastasis suppressors, AKAP12 and SERPINB5, were included for methylation pattern analysis. Our group has previously investigated these genes and identified a stable methylation pattern for the SERPINB5 promoter while AKAP12 displayed variable methylation [26]. Figure 5A is a graphic representation of the differences in overall methylation intensity and variability of methylation intensity. Differences in the standard deviation of the methylation scores was used as a mathematical basis for the definition of variable or constant methylation patterns: methylation score standard deviation of ≤0.09 was defined as constant methylation, and >0.09 as variable methylation (Figure 5B).

In summary, we identified three types of promoter methylation: 1) Genes with completely unmethylated promoters 2) Genes whose promoters were methylated in most cell lines and that showed highly variable methylation intensity (methylation score standard deviation >0.09), dubbed variable methylation. 3) Gene promoters that were methylated in few cell lines and with constant methylation intensity (methylation score standard deviation ≤0.09), dubbed constant methylation.

### Correlation between promoter methylation and loss of mRNA expression

The expression levels of the 4 methylated genes were investigated for differential mRNA expression in correlation to their promoter methylation status. The priorly investigated genes, SERPINB5 (constant methylation pattern) and AKAP12 (variable methylation pattern) were included for this analysis. In the genes with constant methylation (CDH1 and SERPINB5) the significantly methylated cell lines had the lowest mRNA expression across all cell lines. For the variably methylated genes (AKAP12, BRMS1, KiSS-1 and TIMP3), no significant correlations between methylation-score and mRNA expression were detected. Thus, constant methylation patterns exerted influence on mRNA expression in all cases, while all instances of variable methylation patterns had no influence on mRNA expression (Table 1).

**Table 1:** Correlation of methylation patterns to mRNA expression

	**Constant methylation**		**Variable methylation**			
	**CDH1**	**SERPINB5**	**AKAP12**	**BRMS1**	**KiSS-1**	**TIMP3**
Methylation score vs. mRNA expression	P < 0.05	P < 0.05	n.s.	n.s.	n.s.	n.s.

Legend: Constant methylation patterns are correlated to loss of mRNA expression, while variable methylation patterns are not.

### Correlation between metastasis suppressor gene mRNA expression and tumor biology

No significant correlations between MSG mRNA expression and tumor biology were detected. Both invasion and metastasis scores were not correlated to expression of the nine investigated metastasis suppressor genes (Additional file 2: Table S1).

## Discussion

Although aberrant methylation is a frequent epigenetic event for a number of genes in pancreatic cancer, in the present study, promoter methylation was present in only 4 of 9 investigated genes and only resulted in reduced mRNA expression one of them, CDH1.

For further analysis of methylation levels, we included 2 previously investigated, methylated genes: AKAP12 and SERPINB5, increasing the number of methylated genes to 6. For this group of genes we found 2 distinct and mutually exclusive types of methylation, either “constant” or “variable” methylation. Variably methylated genes showed considerable methylation volatility for single CpG sites with methylation levels changing from completely unmethylated to completely methylated several times throughout the sequenced promoter region. The genes in this group were AKAP12, BRMS1, KiSS-1 and TIMP3 -- genes for which there was no correlation between promoter methylation and mRNA expression. Thus, variable methylation, even though often occurring at high levels, did not translate into loss of mRNA expression. This suggests that variable methylation patterns could be a by-product of tumor dedifferentiation and possibly DNMT-1 (DNA methyltransferase 1) overexpression without regulatory links to mRNA-expression. DNMT1 protein expression has been reported to increase progressively during the stages of pancreatic carcinogenesis and was found to be associated with tumor aggressiveness, suggesting that protein overexpression of DNMT1 and ensuing hypermethylation may be involved in multistage pancreatic carcinogenesis -- even though it may not translate into loss of mRNA expression [15]. In contrast, for SERPINB5 and CDH1, the genes with constant methylation patterns, the methylated cell lines showed the lowest mRNA expression across all investigated samples, correlating this pattern of methylation to loss of mRNA expression. These observations suggest that the functional relevance of promoter methylation can be predicted even before carrying out quantitative reverse transcriptase PCR, based only on methylation patterns. While confirmation by qRT-PCR will still be necessary, target selection could be made more efficient through the pre-selection of genes of interest according to their methylation pattern.

Surprisingly, overexpression of some investigated MSGs was found in many tumor cell lines compared to normal pancreatic RNA. For example, BRMS1 demonstrated nearly uniform upregulation in the PDAC cell lines (94%, 17/18 cell lines). This gene was first described in breast cancer and was more recently found to decrease metastatic potential in that tumor entity by upregulating microRNA miR-146 [28]. While we do not know whether miR-146 induction by BRMS1 also occurs in PDAC, it is known that miR-146 is overexpressed in pancreatic cancer vs. both normal pancreas and pancreatitis, suggesting that miR-146 has an oncogenic role in pancreatic cancer [29]. This corresponds to our present findings of nearly uniform upregulation of BRMS1 in PDAC vs. normal tissue. Further investigation of the BRMS1 / miR-146 axis in PDAC appears to be warranted.

We encountered substantial difficulties regarding mRNA expression analysis from in vivo tissues: The recovery of mRNA samples of sufficient quality (RIN > 7.0; tumor cells >80% in microscopic analysis) was not possible due to 2 main reasons: 1) The orthotopic tumor specimens decayed too quickly, likely due to autodigestion before complete permeating of the RNAse inactivating agent, resulting in insufficient RIN values <7.0. 2) The metastases were simply too small for recovery of sufficient sample volume. In addition, it was not possible to control these samples for contamination by normal cells without destroying the sample. Thus, we proceeded without these analyses as they were technically unfeasible.

None of the investigated genes showed any correlation to the metastatic or infiltrative potential of 16 PDAC cell lines *in vivo*. Instead, some were significantly upregulated in the PDAC cell lines vs. normal pancreatic RNA. The present results suggest that most known metastasis suppressor genes do not assume their ascribed roles in PDAC and that some metastasis suppressors even undergo overexpression in this tumor entity. Further, promoter methylation does not seem to be a prominent mechanism of loss of expression for metastasis suppressor genes in PDAC as most genes’ promoter islands showed no methylation at all. Even when methylation did occur, it did not result in loss of mRNA in most cases. Thus, assays for metastasis suppressor gene promoter methylation do not appear to be suitable tools for the detection of PDAC. However, when methylation did induce loss of mRNA expression, a distinct methylation pattern was observed in each case, termed constant methylation. To our knowledge, methylation patterns have not yet been correlated to translation into loss of mRNA expression. Yet methylation patterns may indeed be able to predict whether or not loss of expression occurs when methylation is present. After a first global methylation analysis, information on methylation patterns could help to select genes for further, i.e. functional, analysis. Detailed knowledge of epigenetic alterations and associated molecular mechanisms during pancreatic tumorigenesis and metastasis will broaden the biological understanding of the disease and help devise novel targets for earlier diagnosis and increasingly effective therapies.

## Conclusions

In summary, our results suggest that metastasis suppressor genes may play different roles in PDAC compared to other tumor entities, a hypothesis that must be confirmed by in depth analysis of single genes. In addition, we describe promoter methylation patterns that were correlated to functional relevance: Patterns of constant promoter methylation appear to predict methylation-associated control of gene expression.

## Competing interests

All authors declare that they have no competing interests.

## Authors’ contributions

Study was conceived by JH and WAM. Experiments were carried out by STM and WAM. Statistical analysis was carried out by WAM, STM and JH. Manuscript was written by JH, STM and WAM. All authors read and approved the final manuscript.

## Pre-publication history

The pre-publication history for this paper can be accessed here:

http://www.biomedcentral.com/1471-2407/13/264/prepub

## Supplementary Material

Additional file 1 MSP Gels. Click here for file

Additional file 2: Table S1 Correlation of metastasis suppressor expression to tumor biology. Legend: No significant correlations between metastasis suppressor gene expression and *in vivo *tumor biology were identified. Click here for file
