# Supplementary figures and images for: Epigenetic regulation and role of metastasis suppressor genes in pancreatic ductal adenocarcinoma
Source: BMC Cancer. 2013 May 29;13:264. doi: 10.1186/1471-2407-13-264 (PMC3670210; doi:10.1186/1471-2407-13-264)

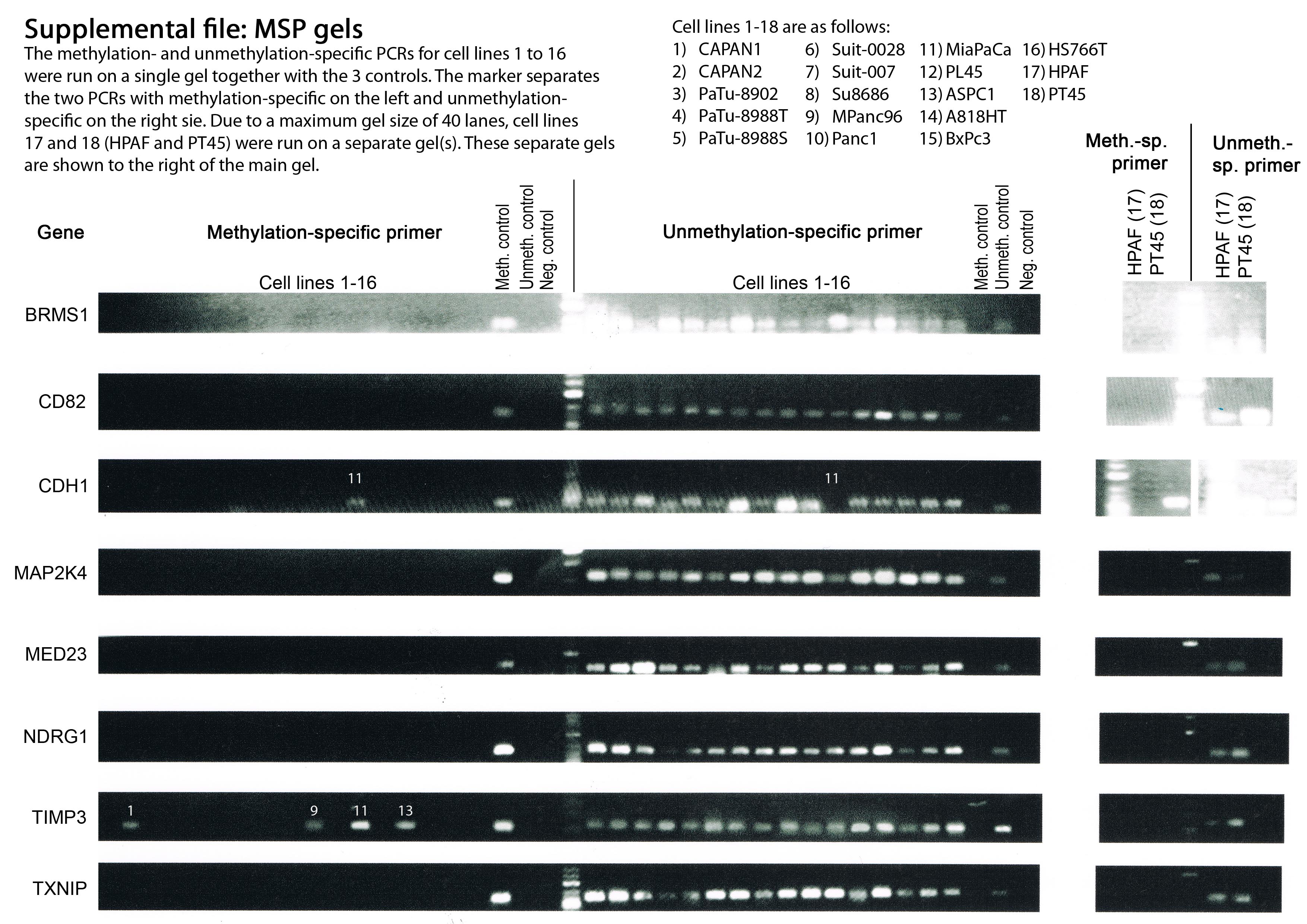

Supplement: Additional file 1 — MSP Gels. [file 1471-2407-13-264-S1.jpeg]
