# Supplementary material for: Epigenetic regulation and role of metastasis suppressor genes in pancreatic ductal adenocarcinoma
Source: BMC Cancer. 2013 May 29;13:264. doi: 10.1186/1471-2407-13-264 (PMC3670210; doi:10.1186/1471-2407-13-264)
Supplement: Additional file 2: Table S1 — Correlation of metastasis suppressor expression to tumor biology. Legend: No significant correlations between metastasis suppressor gene expression and in vivo tumor biology were identified. [file 1471-2407-13-264-S2.doc]

**Supplementary Table 1**

Title: Correlation of metastasis suppressor expression to tumor biology

Legend: No significant correlations between metastasis suppressor gene expression and *in vivo* tumor biology were identified.

|  | BRMS1 | CD82 | CDH1 | KiSS-1 | MED23 | MAP2K4 | NDRG1 | TIMP3 | TXNIP |
| --- | --- | --- | --- | --- | --- | --- | --- | --- | --- |
| Infiltration score | 0,70 | 0,37 | 0,46 | 0,14 | 0,59 | 0,31 | 0,18 | 0,27 | 0,75 |
| Metastasis score | 0,35 | 0,08 | 0,20 | 0,14 | 0,92 | 0,23 | 0,41 | 0,16 | 0,69 |
